# Dual-Facets Emissive Quantum-Dot Light-Emitting Diode Based on AZO Electrode

**DOI:** 10.3390/ma15030740

**Published:** 2022-01-19

**Authors:** Jing Chen, Qianqian Huang, Wei Lei

**Affiliations:** School of Electronic Science and Engineering, Southeast University, Nanjing 210096, China; hqqstudy@126.com (Q.H.); lw@seu.edu.cn (W.L.)

**Keywords:** quantum dot, light-emitting diode, dual-facets emission

## Abstract

We report on a green, dual emissive quantum-dot light-emitting diode (QLED) using alumina (Al)-doped ZnO (AZO) to adjust the band offset between the cathode and QD-emitting layers. The dual emissive QLED structure was designed by enhancing the efficient hole injection/transfer and slowing down the electron injection/transfer from AZO to the QD. The QLEDs presented a maximum luminance of 9450 cd/m^2^, corresponding to a power efficiency of 15.7 lm/W, a current efficiency of 25.5 cd/A, as well as a turn-on voltage of 2.3 V. It is worth noting that the performance of the dual emissive QLED is comparable to that of a single emissive QLED. Therefore, there is a 1.3-fold enhancement in the performance of the QLED based on the AZO cathode due to the balanced charge injection/transfer.

## 1. Introduction

Since the first report of the quantum-dot light-emitting diode (QLED), it has attracted increasingly more attention for merits of their properties, such as high photoluminescence (PL), quantum yield (QY), tunable colors, saturated color emission, narrow emission with full-width at half-maximum (FWHM), solution processability, and process compatibility [1,2,3,4,5]. QLEDs can be expanded to high-volume applications toward commercialization, such as liquid-crystal display backlights [6] and solid-state displays and lighting [7,8,9].

In order to fabricate large-area and low-cost optoelectronic devices, there is an urgent need to develop transparent materials for electrodes with a lower sheet resistance and higher transparency. Recently, for commonly used optoelectronic devices, indium tin oxide (ITO) film can be sputtered at a high temperature of more than 300 °C for limited substrates such as the frequently utilized materials for electrodes. As the electro-optical properties of ITO films can be critically affected by the activation of Sn dopants, it is difficult to fabricate high-quality ITO film without any postprocess, such as heating the substrate or post-annealing [10]. In addition, the high cost of indium (In) as the main component in ITO is a critical problem for ITO electrodes, which limits the large-scale production availability. As a result, it is a requirement to develop alternative, cheap, and transparent materials for electrodes with properties comparable to ITO electrodes. Recently, group III elements, such as Al, Ga, and In-doped ZnO electrodes, have been considered as indium-free and cheaper anode materials for optoelectronic devices, due to the lower price of ZnO materials [11,12]. However, the performance of optoelectronic devices based on Al-doped ZnO (AZO) or Ga-doped ZnO (GZO) electrodes is not comparable, due to the lower conductivity of electrodes [13]. There are some studies in the literature on fully transparent QLEDs based on graphene anodes [14,15,16]. As the WF of the ITO anode is 4.7 eV, which is lower than that of the doped graphene film (5.1 eV), it is closer to the HOMO of PEDOT of 5.0 eV. This may result in a better hole injection between the doped graphene and PEDOT layer, leading to a higher efficiency. The maximum luminescence, the turn-on voltage, and the maximum current efficiency of the QLEDs based on the graphene anode measured from the ITO side are about 30 cd/m^2^ (4 V) and 0.32 cd/A (at 6 V), respectively.

However, reports about QLEDs based on AZO cathodes are rare. It has been reported that AZO electrodes have a better ohmic contact with ZnO NPs than ITO [17]. As a result, it is considered in the design of dual emissive devices based on AZO cathodes, which is deposited on ZnO NPs.

In this study, we report a green dual-facets emissive QLED by usage of AZO as the electrode. Attributed to the more favorable alignment of energy positions, our device exhibits dual-facets emission with a sub-gap turn-on voltage of 2.3 V, a maximum electroluminance intensity of 9450 cd/m^2^, and a power efficiency of 15.7 lm/W.

## 2. Materials and Methods

Preparation of QDs: the synthesis route for green CdZnSeS QDs followed a modified method previously reported [18]. The QDs were eventually dispersed in hexane at a concentration of 20 mg/mL for further devices.

Preparation of devices: the process of fabrication QLEDs followed the previous report [19], except that 150 nm of AZO was sputtered on the top by using an AZO target (Al_2_O_3_-doped ZnO) at a constant direct current power of 100 W with an Ar flow ratio of 20 sccm and working pressure of 5 mTorr on top of the ZnO ETL. The area was 2 × 2 mm^2^. For comparison, a 150 nm aluminum (Al) cathode was thermally evaporated under high vacuum (4 × 10^−6^ Torr) on the upper device, followed by post-annealing at 80–100 °C for 20–30 min. The devices were named samples A and B for single and dual emissive QLEDs, respectively.

Characterization for material and device: The thicknesses of the films within the device were measured by using cross-sectional scanning electron microscopy (SEM). The current density–voltage (J–V) characteristics were measured with a Keithley-2400 source-meter. The absorption and photoluminescence (PL) spectra were measured by UV-visible and FS5 spectrophotometers, respectively. A Cs-corrected high-resolution transmission electron microscope (HRTEM, Titan 80–300, FEI, Gaithersburg, MD, USA) was operated to determine the size and morphology of the QDs, which was carried out operating at an acceleration voltage of 300 kV. The TEM to record the images was equipped with a multiscan charge-coupled device (CCD) camera system (Model 894, Gatan, Pleasanton, CA, USA). X-ray powder diffraction patterns of the QDs were collected by a Bruker D5005 diffractometer using Cu K_α_ radiation (1.5404 Å). The energy-dispersive X-ray (EDX) spectra of QDs were obtained by a Si-Li detector of Oxford INCA energy attached on the main body of the TEM.

## 3. Results

**Figure 1:**
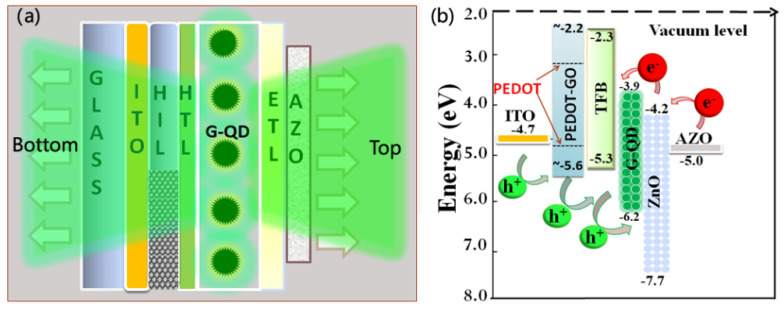
(**a**) A schematic of our device structure and (**b**) corresponding energy levels.

The schematic structure and corresponding energy level for the dual emissive device are displayed in Figure 1a,b, respectively. The dual emissive device contained the structure of ITO/PEDOT:PSS-GO/TFB/QD/ZnO/AZO. A patterned ITO acts as the anode, PEDOT:PSS-GO stands for graphene-doped poly(ethylenedioxythiophene):polystyrene sulphonate (PEDOT:PSS-GO) as the HIL, poly[(9,9-dioctylfluorenyl-2,7-diyl)-co-(4,4-(*N*-(4-sec-butylphenyl)) diphenylamine)] is denoted by TFB as the HTL, the QD is formed with 4 to 5 closely packed monolayers as the emissive layer (EM), ZnO is the ETL, and AZO (for sample B) is the cathode.

**Figure 2:**
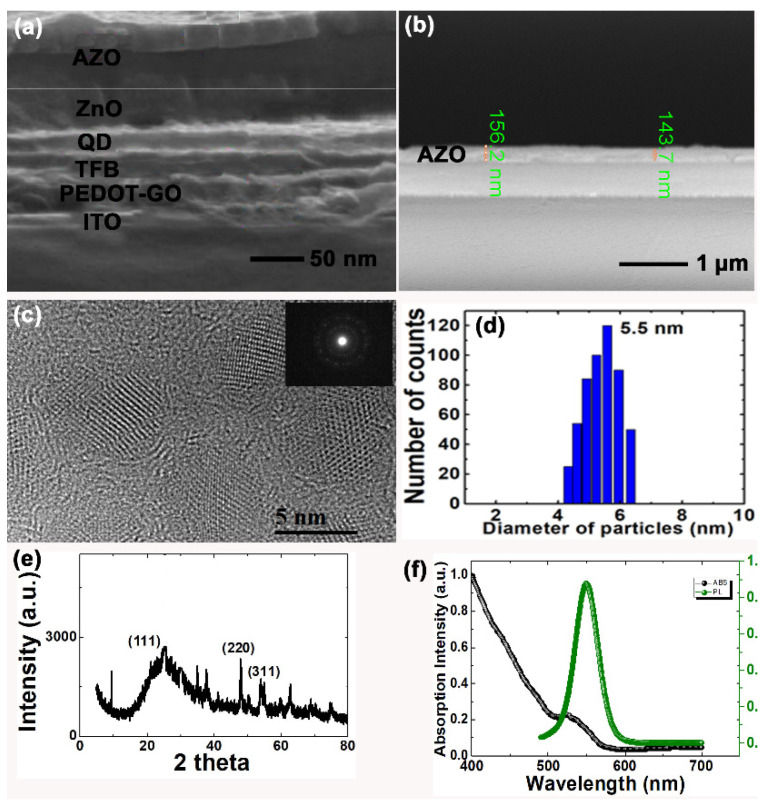
Cross-sectional SEM image of (**a**) QLED vertical structure and (**b**) AZO on glass; (**c**) HRTEM image and diffraction patterns of QDs; (**d**) size distribution; (**e**) XRD patterns; (**f**) absorption and PL spectra of QDs.

The QLED structure was designed to achieve efficient hole injection because the work function of PEDOT-GO was estimated as 5.66 eV, showing a down-shifting in the valence band edge up to 0.66 eV compared to that of pristine PEDOT (at 5.0 eV) [19,20]. At the same time, it effectively slows down the electrons from the electrodes to the QD layer because an injection step of 0.8 eV exists for the injection of electrons from AZO (5.0 eV) to the conduction band (CB) of the QD layer as the ZnO has an electron affinity of 4.2 eV, higher than that from the work function of Al (4.3 eV). On the other hand, it can block holes that pass through the ETL because of the band offsets of the constituent layers (Figure 2b). The difference between the single and dual emissive devices is the output emission direction within the films. For single-facet emission, the anode is ITO, the cathode is Al, and light can only output from the anode side; in our designed dual emissive structure, the refractive index for each layer, as shown in Figure S1a (Supporting Information), allows light emission output toward the cathode and anode directions. Figure S1b (Supporting Information) shows the energy density distributions for normal emission in the dual emissive structure. The optimum dipole positions to maximize forward light output are 30 nm and 150 nm from the anode for ITO and AZO cathodes, respectively.

From the cross-sectional SEM image, the thickness for each layer within QLEDs can be roughly calculated. It can be estimated that PEDOT-GO as the HIL is 50 nm, the TFB layer is 40 nm, the QD emissive layer is 30 nm, the ZnO layer is 40 nm, and AZO (sample B) as the cathode is 150 nm. The thickness of the single layer of AZO deposited on glass can be confirmed as around 150 nm (Figure 2b).

As shown in Figure 2c, the HRTEM image indicates that CdZnSeS QDs without the core–shell structure were uniformly dispersed in hexane with an average diameter of 5–6 nm. The diffraction patterns indicate the good crystallization of QDs. Statistically analyzed from the HRTEM image in Figure 2d, we can calculate the mean size of CdZnSeS QDs as 5.54828 nm, with a standard deviation of 0.83558 nm. The QDs were quite uniform. The XRD patterns show the zinc-blend structure with crystalline facets of (111), (220), and (311) in Figure 2e. The EDX spectrum in Figure S2 demonstrates that the chemical composition of the gradient of QDs was CdZnSeS. Cu was ordinated from the copper substrate, while C, O, and Si elements came from the atmosphere.

Figure 2f shows the UV-vis absorption and PL spectra of CdZnSeS QDs utilized in this study. The absorption curve clearly indicates the first excitonic transition peak located at 536 nm. As a result, the band gap (E_g_) of QDs can be calculated by the x-axis value according to the absorption excitonic transition peak [21]. It was estimated as 2.3 eV for this variety of green-emission CdZnSeS QDs. The PL curve reveals a Gaussian-shaped peak positioned at 550 nm with a narrow FWHM of ~30 nm. By using a Rhodamine 6G reference, the QY of CdZnSeS QDs can be calculated as 80%.

**Figure 3:**
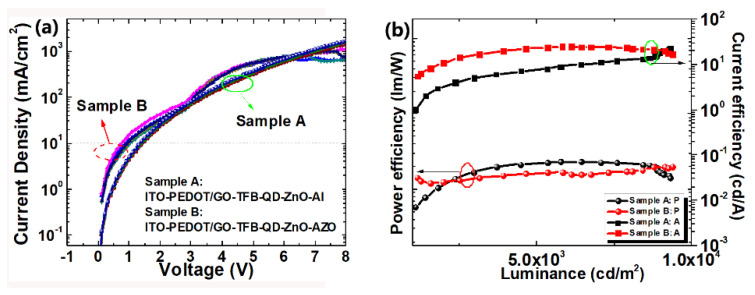
(**a**) The current density–voltage (J–V) and (**b**) brightness and power efficiency (ηA-L-ηp) of sample A and B under forward bias.

Figure 3a shows the current density–voltage (J–V) for sample A and B under forward bias from 0 to 8 V. The performance parameters are summarized in Table 1. The devices exhibited an average turn-on voltage of 2.3 V for sample B and 2.7 V for sample A. Compared to sample A, the turn-on voltage of sample B clearly decreased; on the one hand, this is primarily due to the reduced injection and transfer barrier due to graphene doping; on the other hand, it slows down the electron transfer rate from AZO to QDs; therefore, it brings in a more balanced hole and electron transfer and recombination. In addition, it is observed that the turn-on voltage of the dual emissive QLED can be as low as the energy barrier for the green QD (2.3 eV), indicating the direct exciton recombination mode within the QD during the working process. Figure 3b presents relationship between the brightness and power efficiency (η_A_-L-η_p_) of sample A and B for driving voltages varying from 0 to 8 V. A maximum brightness, current efficiency, and power efficiency of 9450 cd/m^2^, 25.5 cd/A, and 15.7 lm/W can be obtained for sample B, respectively; and a maximum brightness, current efficiency, and power efficiency of 9300 cd/m^2^, 23.0 cd/A, and 12.5 lm/W can be obtained for sample A, respectively.

To characterize the electron injection/transfer efficiency of the cathode, electron-only devices with ITO/QDs/ZnO/Al and ITO/QDs/ZnO/AZO were fabricated and measured. Meanwhile, a hole-only device (ITO/PEDOT-GO/TFB/AZO) was also fabricated for comparison, as shown in Figure S3 (Supporting Information). Devices based on AZO exhibited a lower electron current than that of devices with Al. This is because it slows down the electron transfer rate from AZO to QDs. It turns out that the current density in the electron-only device with AZO becomes similar to that in the hole-only device, indicating a more balanced hole and electron transfer and recombination in the QLED device.

**Figure 4:**
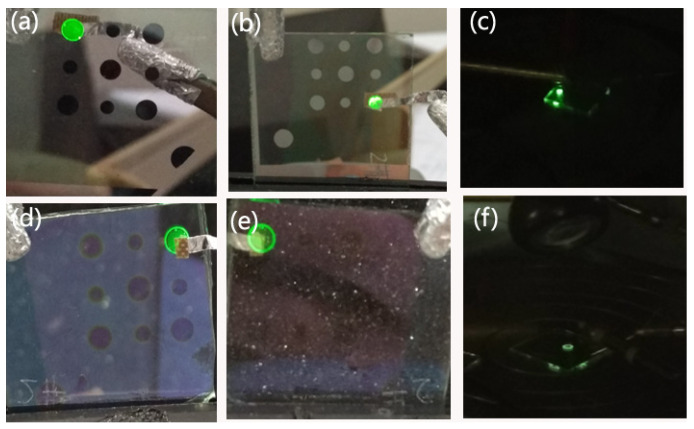
Bottom and top view of sample A (**a**,**b**) and sample B (**d**,**e**) and corresponding probe station test (**c**,**f**).

Therefore, compared to the Al cathode, electrons and holes can be more balanced injected from the AZO cathode, except that the AZO is transparent and can realize dual emission. Demonstration photos of sample A and sample B are shown in Figure 4. Compared to that of sample A, sample B looks much more transparent from the top and bottom view. Though the value of luminance intensity of sample B is comparable to that of sample A, without Al reflection, the luminance looks lower than that of sample A.

## 4. Conclusions

In this work, a dual emissive QLED was demonstrated using AZO as the cathode. Attributed to the downward shift in the HOMO level of the doped HIL and the larger injection step between AZO and QD to decelerate the electron transfer rate, the hole and electron transfer and recombination processes were largely balanced, which played a key role in boosting the performance of the QLED. Finally, the performance of the QLED could also be enhanced with a lowered turn-on voltage and boosted electroluminance intensity. Compared to that with the Al cathode, we found a 1.3-fold improvement in the performance of the QLED, as well as the dual emission based on the AZO cathode. The turn-on voltage could be as low as the bandgap energy for the green QDs, proving the direct exciton recombination method within the QDs. For the next step, the technique can be extended to energy band engineering for blue- or white-light QLEDs by the AZO cathode.

## Figures and Tables

**Table 1:** Parameters obtained from J–V and η_A_-L-η_p_ curves.

	Turn-On Voltage (V)	Maximum Brightness (cd/m^2^)	Power Efficiency (lm/W)	Current Efficiency (cd/A)
Sample A	2.7	9300	12.5	23.0
Sample B	2.3	9450	15.7	25.5

## Data Availability

Not applicable.

## Supplementary Materials

The following supporting information can be downloaded at: https://www.mdpi.com/article/10.3390/ma15030740/s1, Figure S1: (a) the refractive index for each layer (b) Energy density as a function of distance from QD for du-al emissive device, Figure S2: elements analysis results (a) from EDX spectrum (b), Figure S3: Current density as a function of voltage for the electron only and hole-only devices.
