# Supplementary material for: Dual-Facets Emissive Quantum-Dot Light-Emitting Diode Based on AZO Electrode
Source: Materials (Basel). 2022 Jan 19;15(3):740. doi: 10.3390/ma15030740 (PMC8836426; doi:10.3390/ma15030740)
Supplement: Supplementary file 1 [file materials-15-00740-s001.zip › materials-1516031-supplementary.pdf]

materials

MDPI

Supporting information

Dual facets emissive quantum dot light emitting diode based on AZO electrode

Jing Chen *, Qianqian Huang and Wei Lei

School of Electronic Science and Engineering, Southeast University, Nanjing 210096, China; chenjing@seu.edu.cn

* Correspondence: chenjing@seu.edu.cn;

Abstract: We report on a green, dual emissive quantum dot light emitting diode (QLED) using alumina (Al) doped ZnO (AZO) to adjust the band offset between the cathode and QD emitting layers. The dual emissive QLED structure was designed by enhancing efficient hole injection/transfer and slowing down the electron injection/transfer from AZO to QD. The QLEDs present a maximum luminance of 9450 cd/m^2, corresponding to a power efficiency of 15.7 lm/W, current efficiency of 25.5 cd/A as well as a turn on voltage of 2.3 V. It is worth noting that the performance of dual emissive QLED is compatible to that of single emissive QLED. Therefore, there is a 1.3-fold enhancement in the performance of the QLED based on AZO cathode due to the balanced charge injection/transfer.

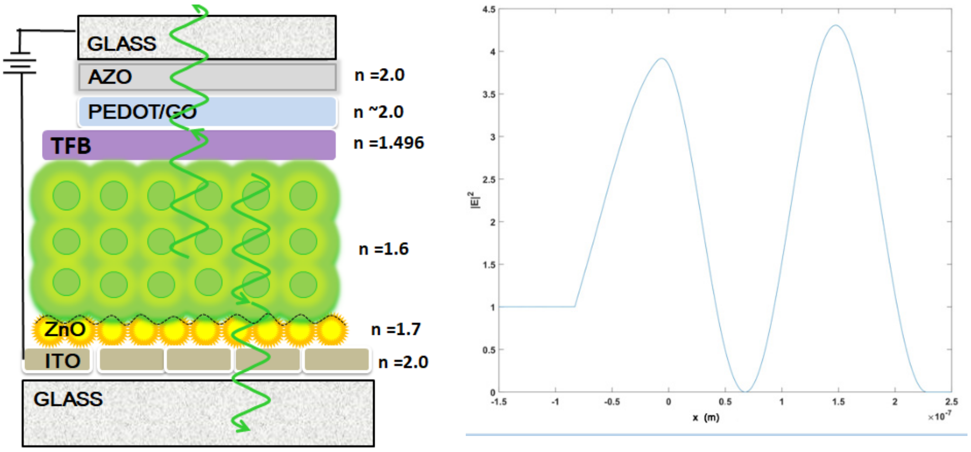

(**a**) (**b**)

Figure S1. (**a**) the refractive index for each layer (**b**)Energy density as a function of distance from QD for dual emissive device.

For single facet emission, anode is ITO, cathode is Al, light can only output from anode side; in our designed dual emissive structure, the refractive index for each layer as Figure S2(a), it allows light emission output towards cathode and anode directions. Figure S2(b) shows the energy density distributions for normal emission in dual emissive structure. The optimum dipole positions to maximize forward light output are 30 nm and 150 nm from the anode for ITO and AZO cathodes, respectively.

Element	Weight%	Atomic%
C K	22.60	57.15
O K	2.82	5.35
Si K	2.47	2.67
S K	8.25	7.81
Cu K	40.16	19.20
Zn K	6.34	2.95
Se L	1.47	0.56
Cd L	15.89	4.29
Totals	100.00	

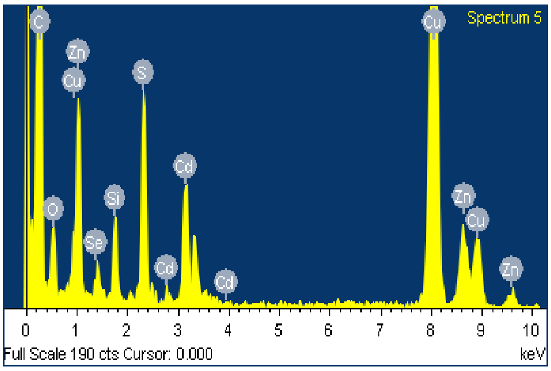

(**a**) (**b**)

Figure S2. elements analysis results(**a**) from EDX spectrum(**b**).

XRD patterns indicate zinc-blend structure for CdZnSeS QDs with crystalline facets of (111)、(220) and (311). It is demonstrated that the chemical compositions of gradient of QDs as CdZnSeS. Cu is ordinated from copper substrate. C、O、Si elements are from atmosphere.

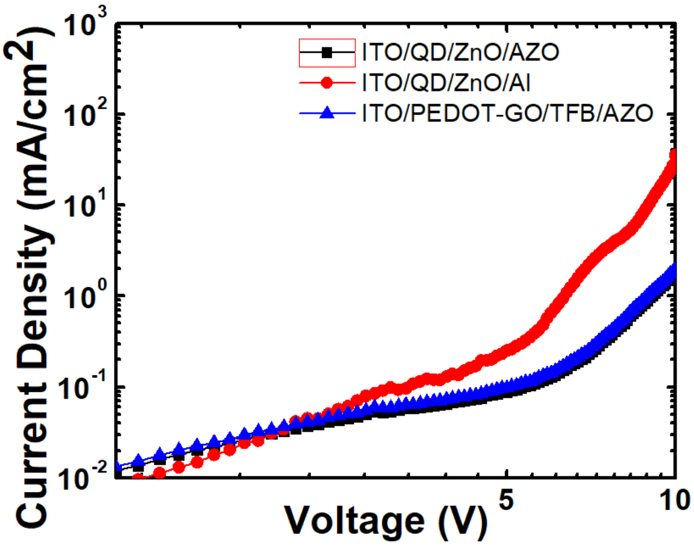

Figure S3. Current density as a function of voltage for the electron only and hole-only devices.

To characterize the electron injection/transfer efficiency of the cathode, electron-only devices with ITO/QDs/ZnO/Al and ITO/QDs/ZnO/AZO were fabricated and measured. Meanwhile, hole only device (ITO/PEDOT-GO/TFB/AZO) were also fabricated for comparison as shown in Figure S3. Devices with AZO as cathode exhibited the lower electron current than that of devices with Al. This is because it slows down the electron transfer rate from AZO to QDs. It turns out that the current density in the electron-only device with AZO becomes similar to that in the hole-only device, indicating a more balanced hole and electron transfer and recombination in the QLED device.
